# Defining the metabolic requirements for the growth and colonization capacity of *Campylobacter jejuni*

**DOI:** 10.3389/fcimb.2014.00137

**Published:** 2014-09-29

**Authors:** Dirk Hofreuter

**Affiliations:** Hannover Medical School, Institute for Medical Microbiology and Hospital EpidemiologyHannover, Germany

**Keywords:** *Campylobacter jejuni*, intermediary metabolism, amino acid catabolism, peptide catabolism, respiration, colonization, intracellular survival

## Abstract

During the last decade *Campylobacter jejuni* has been recognized as the leading cause of bacterial gastroenteritis worldwide. This facultative intracellular pathogen is a member of the Epsilonproteobacteria and requires microaerobic atmosphere and nutrient rich media for efficient proliferation *in vitro*. Its catabolic capacity is highly restricted in contrast to *Salmonella* Typhimurium and other enteropathogenic bacteria because several common pathways for carbohydrate utilization are either missing or incomplete. Despite these metabolic limitations, *C. jejuni* efficiently colonizes various animal hosts as a commensal intestinal inhabitant. Moreover, *C. jejuni* is tremendously successful in competing with the human intestinal microbiota; an infectious dose of few hundreds bacteria is sufficient to overcome the colonization resistance of humans and can lead to campylobacteriosis. Besides the importance and clear clinical manifestation of this disease, the pathogenesis mechanisms of *C. jejuni* infections are still poorly understood. In recent years comparative genome sequence, transcriptome and metabolome analyses as well as mutagenesis studies combined with animal infection models have provided a new understanding of how the specific metabolic capacity of *C. jejuni* drives its persistence in the intestinal habitat of various hosts. Furthermore, new insights into the metabolic requirements that support the intracellular survival of *C. jejuni* were obtained. Because *C. jejuni* harbors distinct properties in establishing an infection in comparison to pathogenic Enterobacteriaceae, it represents an excellent organism for elucidating new aspects of the dynamic interaction and metabolic cross talk between a bacterial pathogen, the microbiota and the host.

## Introduction

Campylobacteriosis has emerged as major bacterial food-borne disease in industrialized countries in recent years (Epps et al., 2013). Mainly *C. jejuni* is associated with acute *Campylobacter* enteritis in humans causing more than 80% of the registered *Campylobacter* infections. Predominant sources of infections are contaminated meat (especially chicken), raw milk and water. The clinical manifestations of *Campylobacter* enteritis are indistinguishable from *Salmonellosis* and range from mild watery to severe, inflammatory and bloody diarrhea accompanied with abdominal pain and fever (Allos, 2001). Such variations in the disease outcome might correlate with the well documented different virulence potential of individual *C. jejuni* isolates and are possibly linked to dissimilarities in motility and surface structures involved in the direct interaction with the host. These variable structures include the lipooligosaccharide, the capsule and the glycosylation pattern of the flagellin (Wilson et al., 2010). Interestingly, not only variable surface structures but also metabolic traits are highly variable between *C. jejuni* isolates: Genes supporting the oxygen-independent respiration and the catabolism of amino acids and peptides are particular over-represented in robust-colonizing strains compared to poor-colonizing strains (Ahmed et al., 2002; Hofreuter et al., 2006; Hepworth et al., 2007; Seal et al., 2007; Hiett et al., 2008). Such observations suggest that the physiological properties of *C. jejuni* play a crucial role for its pathogenesis. While some aspects of *C. jejuni* pathogenesis have been described in detail previously (Young et al., 2007; Janssen et al., 2008; van Putten et al., 2009; Gilbreath et al., 2011; Szymanski and Gaynor, 2012), this review specifically summarizes our increasing knowledge about the *in vitro* and *in vivo* metabolism of *C. jejuni* and its impact on the virulence and colonization process of this important pathogen.

## General physiological properties of *C. jejuni*

Though *C. jejuni* shows fastidious growth characteristics *in vitro* and easily looses viability as well as culturability, it resides as a commensal in a wide range of diverse animal hosts (e.g., chicken, cattle, sheep, goat, dog, duck and pig) and can be isolated from various environmental sources or refrigerated foods. This life style implies that *C. jejuni* is able to resist varying temperatures, oxygen concentrations, pH values, osmotic environments and nutrient availabilities as reviewed comprehensively elsewhere (Park, 2002; Murphy et al., 2006). *C. jejuni* belongs to the group of thermophilic *Campylobacter* species that grow preferentially between 42°C and 37°C, but do not proliferate below 30°C (Penner, 1988). It was suggested that the absence of cold-shock proteins might be responsible for the inability of this pathogen to grow at lower temperatures (Hazeleger et al., 1998). Nonetheless, *C. jejuni* shows respiration and ATP generation at temperatures as low as 4°C, maintaining its metabolic activities at low temperatures for an extended time period (Hazeleger et al., 1998). *C. jejuni* survives at 4°C even better than at 25°C (Blaser et al., 1980), which leads to the common problem that refrigerated meat contaminated with *C. jejuni* during the slaughter process represents a particular frequent source of *C. jejuni* infections (Bhaduri and Cottrell, 2004). While *C. jejuni* is able to withstand low temperature, atmospheric oxygen concentration affects its viability dramatically. The microaerophilic nature of *C. jejuni* requires an atmosphere with reduced oxygen and elevated carbon dioxide concentrations for its efficient cultivation *in vitro*: Variable oxygen tolerance has been described for *C. jejuni* isolates, but gas mixtures of 5% oxygen, 10% carbon dioxide and 85% nitrogen provide optimal cultivation conditions for most *C. jejuni* isolates (Bolton and Coates, 1983). *C. jejuni* has with about 800 bacteria a lower infective dose than *Salmonella* Typhimurium or pathogenic *Escherichia coli* strains (Black et al., 1988; Kothary and Babu, 2001), indicating that *C. jejuni* is well adapted to survive the harsh, acidic environment of the human stomach. Moreover, it demonstrates the proficiency with which *C. jejuni* is able to multiply in the gastrointestinal tract of humans and to consume the nutritional sources present in the host intestine in order to overcome the microbiota-mediated colonization resistance. Therefore, characterizing the metabolic properties of *C. jejuni* that allows this successful human pathogen and widespread animal commensal to thrive in its diverse hosts has gained increasing attention in recent years.

## Finding a nutritional niche: the low-carb, high-protein diet of *C. jejuni*

### The non-glycolytic nature of *C. jejuni*

*C. jejuni* is a chemoheterotrophic bacterium with metabolic properties that clearly distinguish it from other enteropathogenic bacteria. Most striking is its restricted carbohydrate catabolism: Early physiological studies examining the substrate utilization of *Campylobacter* identified its incapability to use glucose and other carbohydrates as growth substrates, and since then *Campylobacter* is generally considered to be a non-saccharolytic bacterium. These observations were supported by genome sequence analysis (Parkhill et al., 2000; Velayudhan and Kelly, 2002; Gundogdu et al., 2007) and recent growth-independent BIOLOG phenotype microarray analyses based on the tetrazolium redox dye chemistry that allows monitoring the respiratory activity of metabolically active cells (Bochner, 2009). This approach confirmed that pentoses and hexoses like glucose, fructose, galactose, rhamnose and the disaccharides lactose, maltose, trehalose and sucrose do not enhance the respiratory activity of *C. jejuni* (Line et al., 2010; Gripp et al., 2011; Muraoka and Zhang, 2011). However, these studies did reveal that certain strains like *C. jejuni* NCTC 11168 are capable to catabolize fucose in contrast to other isolates. The observed metabolic diversity is explained by the occurrence of a 9 kb genomic island, comprised in *C. jejuni* NCTC 11168 of the open reading frames Cj0480 to Cj0490, which are absent in *C. jejuni* 81–176 (Hofreuter et al., 2006; Muraoka and Zhang, 2011; Stahl et al., 2011). This gene region encodes for a putative fucose permease FucP (Cj0486) with homology to major facilitator superfamily (MFS) transporters. It was demonstrated that FucP enhanced the growth of *C. jejuni* NCTC 11168 when cultivated in chemically defined media containing 25 or 50 mM fucose as an additional carbon and energy source (Muraoka and Zhang, 2011; Stahl et al., 2011). The catabolic pathway of fucose in *fucP*-positive *C. jejuni* strains is not clear yet. Preliminary data suggest that the fucose utilization does not occur as observed for other intestinal bacteria like *E. coli* or *Bacteroides* species but as described in *Xanthomonas campestris* generating pyruvate and lactate (Stahl et al., 2011). Both end products are utilized by *C. jejuni* and efficiently promote its *in vitro* growth (Mendz et al., 1997; Velayudhan and Kelly, 2002; Thomas et al., 2011). Interestingly, no secreted fucosidase enzymes that cleave fucose residues from glycosylated host proteins have been described for the fucose-catabolizing *C. jejuni* strains. This suggests that *fucP* positive *C. jejuni* isolates might rely on the fucose released from intestinal mucins and host glycans by commensal bacteria like *Bacteroides thetaiotaomicron* as demonstrated for enterohaemorrhagic *Escherichia coli* (Pacheco et al., 2012). Alternatively, the activity of an induced host fucosidase could make free fucose available for *C. jejuni* similar as described for the human fucosidase FUCA2 of cultured human gastric and pancreatic adenocarcinoma cells upon infection with *Helicobacter pylori* (Liu et al., 2009).

The incapability to catabolize glucose distinguishes *C. jejuni* from many other gastrointestinal pathogens like *Salmonella* Typhimurium, enteropathogenic *E. coli* or *Listeria monocytogenes* (Dandekar et al., 2012; Fuchs et al., 2012) but also from its close relative *H. pylori* (Mendz et al., 1993). *C. jejuni* and *H. pylori* have an interrupted Embden–Meyerhof–Parnas (EMP) pathway because they lack the glycolytic enzyme phosphofructokinase, which catalyzes the phosphorylation of fructose-6-phosphate to fructose-1,6-bisphosphate. However, *H. pylori* possesses a complete pentose phosphate pathway and an Entner–Doudoroff pathway (Doig et al., 1999) that enable the catabolism of glucose to pyruvate (Mendz et al., 1994), while *C. jejuni* does not encode for the glucose-6-phosphate dehydrogenase (EC 1.1.1.49) and the 6-phosphogluconolactonase (EC 3.1.1.31) of the oxidative pentose phosphate pathway branch but harbors all enzymes of the reductive branch of the pentose phosphate pathway (Parkhill et al., 2000; Fouts et al., 2005). Consequently, gluconeogenesis seems to be required for the biosynthesis of glucose and its derivatives that are precursor substrates for the LPS and capsule biosynthesis as well as the N- and O-glycosylation of various secreted proteins (Karlyshev et al., 2005). The genome of *C. jejuni* harbors all enzymes necessary for gluconeogenic synthesis of glucose from phosphoenolpyruvate (PEP) (Parkhill et al., 2000), but gluconeogenesis has not been experimentally proven yet. Only few studies have characterized the intermediary metabolism of *C. jejuni* and the anaplerotic reactions fueling gluconeogenesis, but it was demonstrated that the PEP carboxykinase PckA (Cj0932c), the pyruvate kinase Pyk (Cj0392c) and the pyruvate carboxylase (PycA, Cj1037c; PycB, Cj0933c) comprise a crucial metabolic junction between catabolism and anabolism in *C. jejuni* (Velayudhan and Kelly, 2002). These enzymes are required for the oxaloacetate—PEP—pyruvate conversion that comprises a metabolic triangle, which plays the central role in controlling the carbon flow by connecting the tricarboxylic acid (TCA) cycle with the lower portion of the EMP pathway (Sauer and Eikmanns, 2005). Interestingly, *C. jejuni* does not encode for a PEP carboxylase catalyzing the generation of oxaloacetate from PEP and is lacking a PEP synthase to mediate the first ATP-consuming gluconeogenic reaction that synthesizes PEP from pyruvate. Consequently, the PEP carboxykinase PckA, catalyzing the synthesis of PEP by the decarboxylation of oxaloacetate, plays an important role in the intermediary metabolism of *C. jejuni* (Velayudhan and Kelly, 2002). Further studies are needed to identify the preferred substrates fueling the lower portion of the EMP pathway and to investigate how the intermediary metabolism is regulated and fine-tuned in *C. jejuni* in order to establish a balance between anabolism and catabolism.

### Organic acids and amino acids fuel the growth of *C. jejuni*

Because the gluconeogenesis seems to play a crucial role in the physiology of *C. jejuni*, it raises the question of which substrates are efficiently utilized by this pathogen to fuel its intermediary metabolism and cope with its necessities for carbohydrate, lipid and protein biosynthesis. Various studies using the API test system (Elharrif and Megraud, 1986), the detection of CO_2_ release upon incubation of *C. jejuni* with ^14^C-labeled substrates (Westfall et al., 1986), substrate oxidation experiments with an oxygen electrode system (Mohammed et al., 2004) and *in vitro* growth experiments (Velayudhan and Kelly, 2002; Hinton, 2006; Guccione et al., 2008; Wright et al., 2009) revealed that *C. jejuni* catabolizes organic acids like lactate, pyruvate, acetate and intermediates of the TCA cycle as well as a restricted number of amino acids. The importance of organic acids for the proliferation of *C. jejuni* was further demonstrated by studies that identified pyruvate, 2-oxoglutarate, fumarate, succinate, malate and lactate as chemoattractants of *C. jejuni* (Hugdahl et al., 1988; Vegge et al., 2009).

The L- and D-lactate catabolism of *C. jejuni* has been described in detail: L-lactate is taken up by *C. jejuni* NCTC 11168 with the symport of protons by the transporter protein Cj0076c that belongs to the lactate permease LctP family (TC 2.A.14). In addition, other not yet identified transporter proteins are probably involved in the uptake of L- and D-lactate (Thomas et al., 2011). The imported L-lactate is subsequently oxidized to pyruvate, which itself is a growth substrate of *C. jejuni* (Mendz et al., 1997; Velayudhan et al., 2004) though no pyruvate carrier has been described yet. *C. jejuni* does not harbor a pyruvate dehydrogenase found in other enteropathogenic bacteria but catalyzes the oxidative decarboxylation of pyruvate to acetyl-CoA through a pyruvate:acceptor oxidoreductase (POR; Cj1476c) similar as described for *H. pylori* (St Maurice et al., 2007). However, the POR enzyme of *H. pylori* is composed of the four subunits PorABCD (Hughes et al., 1998), whereas the pyruvate-flavodoxin oxidoreductase of *C. jejuni* is comprised of one polypeptide with four functional domains. It is conserved in other *Campylobacter* species and has homologs in host-associated bacteria like *Fusobacterium nucleatum*, *Fusobacterium necrophorum*, *Cetobacterium somerae* or *Sebaldella termitidis*, as well as environmental bacteria such as *Psychrilyobacter atlanticus* and *Orenia marismortui*. Interestingly, the pyruvate-flavodoxin oxidoreductase of *C. jejuni* is responsible for its sensitivity against nitazoxanide, an antiprotozoal drug that inhibits PORs but not pyruvate dehydrogenases (Hoffman et al., 2007). The POR catalyzed generation of acetyl-CoA both fuels the TCA cycle of *C. jejuni* and is used for fatty acid biosynthesis (Leach et al., 1997; Gundogdu et al., 2007; Kirkpatrick et al., 2009). Furthermore, acetyl-CoA can be converted to acetyl-P and subsequently to acetate by the consecutive activities of the phosphate acetyltransferase Pta (Cj0688) and the acetate kinase AckA (Cj0689). The generated acetate is secreted by a yet unknown transporter during the logarithmic growth phase (Wright et al., 2009). In the late logarithmic growth phase an “acetate switch,” which is well characterized for *E. coli* (Wolfe, 2005), could be observed for *C. jejuni*: The previously secreted acetate is taken up and can be used as a growth substrate through the conversion to acetyl-CoA by the acetyl-CoA synthetase ACS (Cj1537c) (Wright et al., 2009).

It is well established that the utilization of amino acids plays an important role in fueling the central metabolism of *C. jejuni*. Strikingly, however, only few glucogenic amino acids are degraded by this pathogen and support its proliferation, and some amino acids such as arginine and lysine have been described as chemorepellents (Rahman et al., 2014). For most *C. jejuni* isolates the growth-promoting amino acids are aspartate, glutamate, proline and serine (Leach et al., 1997; Guccione et al., 2008; Hofreuter et al., 2008). This finding is in agreement with the metabolic profiling of supernatants from *C. jejuni* liquid cultures demonstrating a significant depletion of these four amino acids from nutrient-rich brain heart infusion (BHI) or Mueller-Hinton (MH) medium (Guccione et al., 2008; Wright et al., 2009). The utilization of the growth-promoting amino acids in liquid cultures occurs in sequential phases: Aspartate and serine are first catabolized and facilitate the rapid growth of *C. jejuni* followed by the usage of glutamate. Proline seems to be a less-preferred growth substrate of *C. jejuni* because its consumption from the culture medium occurred less rapidly in comparison to the depletion of aspartate, serine and glutamate (Leach et al., 1997; Weingarten et al., 2009; Wright et al., 2009). Accordingly, L-aspartate, L-glutmate and L-serine but not L-proline are effective chemoattractants for *C. jejuni* (Hugdahl et al., 1988; Vegge et al., 2009).

Serine is imported by the high-capacity, low affinity serine transport protein SdaC (Velayudhan et al., 2004), a serine/H^+^ symporter of the hydroxy/aromatic amino acid permease (HAAAP) family TC 2.A.42.2. The *sdaC* gene (*cj1625c*) is organized in an operon with *sdaA* (*cj1624c*) encoding for a serine dehydratase that catalyzes the deamination of serine to pyruvate. SdaA harbors, like various respiratory enzymes of *C. jejuni*, an oxygen-labile [4Fe–4S] cluster that might contribute to the oxygen sensitivity of this pathogen (Velayudhan et al., 2004). Serine utilization seems to be a variable catabolic trait of *C. jejuni* because not all tested isolates were able to grow with this amino acid a sole carbon source (Hofreuter et al., 2008). The molecular basis for this metabolic diversity is not known yet, as *C. jejuni* strains unable to utilize serine have no mutations in the *sdaA* and *sdaC* genes though the serine dehydratase activity was fairly reduced (Hofreuter et al., 2012).

In contrast to serine catabolism, proline utilization seems to be a more conserved metabolic trait of *C. jejuni* as also shown for the usage of aspartate and glutamate (Hofreuter et al., 2008). A directed mutagenesis approach demonstrated that the growth of *C. jejuni* 81–176 with proline is mediated by the PutP transporter and the enzyme PutA (Hofreuter et al., 2012). PutP (CJJ81176_1494) belongs to the Na^+^/solute symporter family TC 2A.21, which is widespread in Gram-positive and Gram-negative bacteria (Jung et al., 2012). It is highly conserved in *C. jejuni* and shows about 80% amino acid identity to respective transporter proteins of *C. coli*, *C. lari*, *C. upsaliensis* and *C. fetus*, whereas no homologs are present in other *Campylobacter* species. In addition, the proline symporter protein (PutP_Cj_) of *C. jejuni* shows 75% identity to the PutP transporter (PutP_Hp_) of the closely related *H. pylori* (Hofreuter et al., 2012). So the PutP_Cj_-mediated proline import might have comparable properties as the PutP_Hp_-catalyzed uptake of proline, which depends entirely on Na^+^ as a coupling ion and binds specifically to L-proline with high affinity (Rivera-Ordaz et al., 2013). The PutA (CJJ81176_1495) enzyme of *C. jejuni* is predicted to possess both proline dehydrogenase and delta-1-pyrroline-5-carboxylate dehydrogenase activities and to use FAD and NADH as cofactors, respectively. It catalyzes the oxidation of the imported proline to glutamate. The *putA* and *putP* genes of *C. jejuni* and other Epsilonproteobacteria compromise an operon structure, whereas the adjacent located *putA* and *putP* genes of Enterobacteria have an inverse orientation (Hofreuter et al., 2012). Furthermore, the PutA protein of *C. jejuni* does not contain the N-terminal DNA-binding domain, which is involved in the repression of *putAP* gene cluster in *Salmonella* Typhimurium during the absence of proline (Ostrovsky de Spicer and Maloy, 1993).

*C. jejuni* takes up glutamtate through an ABC transporter system encoded by the *peb* locus (*cj0919c*-*cj0922c*) harboring genes for two permeases, one ATP-binding protein and one periplasmic substrate-binding protein Peb1A (Cj0921c). The latter has been first described as surface-bound antigen of *C. jejuni* with homology to amino acid-binding proteins like GlnH and HisJ (Pei and Blaser, 1993). Further studies characterized Peb1A as a glutamate- and aspartate-binding protein (Leon-Kempis Mdel et al., 2006; Müller et al., 2007), and an isogenic *C. jejuni peb1A* mutant showed impaired growth with aspartate or glutamate as sole carbon sources (Leon-Kempis Mdel et al., 2006). This phenotype correlated with an abolished glutamate uptake. In addition to the Peb ABC transporter, mutations of the permease PaqP (Cj0467) and the ATPase PaqQ (Cj0469) of the Paq (pathogenesis-associated glutamine) ABC transporter system negatively affected the uptake of glutamate in *C. jejuni* 81–176 (Lin et al., 2009). Imported glutamate is unlikely to be converted directly to 2-oxoglutarate through deamination since *C. jejuni* lacks a glutamate dehydrogenase (EC 1.4.1.2, EC 1.4.1.3, EC 1.4.1.4), which is present in other representatives of the *Campylobacteriaceae*. Instead, glutamate might either be converted to glutamine by the type I glutamine synthetase GlnA (Cj0699c) or is substrate of the aspartate:glutamate transaminase AspB (Cj0762c) catalyzing the generation of aspartate and 2-oxoglutarate from oxaloacetate and glutamate (Guccione et al., 2008). AspB plays an important role in the intermediary metabolism of *C. jejuni* and its efficient proliferation, since the inactivation of *aspB* results in an severe growth defect of the respective mutant in nutrient rich MH or BHI media as well as in defined medium with 20 mM glutamate or serine as sole energy sources (Guccione et al., 2008; Novik et al., 2010).

Besides serine, proline and glutamate catabolism, the utilization of aspartate is crucial for the metabolic fitness of *C. jejuni*. This is reflected by the identification of two chemoreceptors, Tlp1 (Hartley-Tassell et al., 2010) and CcmL (Tlp3) (Rahman et al., 2014), that interact with aspartate. The uptake of aspartate is convoluted and involves various transporters: While the periplasmic binding protein Peb1A interacts with aspartate and participates in its uptake, the *peb1A* mutant still possesses capacity to import aspartate although with a 20-fold reduction (Leon-Kempis Mdel et al., 2006). The direct involvement of the other Peb ABC transporter components like the two Peb permeases and the Peb ATP-binding protein has not been experimentally proven yet. In addition to the Peb1A-mediated aspartate uptake, the C4-dicarboxylate antiporters DcuA (Cj0088) and DcuB (Cj0671) have been shown to transport aspartate into *C. jejuni* under oxygen-limited conditions. Their activities were redundant so only a *dcuA*/*dcuB* double mutant showed reduced growth with aspartate (Guccione et al., 2008). It was suggested that aspartate uptake through the C4-dicarboxylate transport protein DctA (Cj1192) was responsible for the remaining growth of the *dcuA*/*dcuB* double mutant (Guccione et al., 2008). Interestingly, also a *paqP* mutant but not a *paqQ* mutant of the Paq ABC transporter showed a reduced level of aspartate uptake (Lin et al., 2009). The fate of imported aspartate is multifaceted involving central anabolic as well as catabolic pathways of *C. jejuni*: Aspartate represents an efficient carbon and energy source for *C. jejuni* as it directly fuels the TCA cycle by the aspartate ammonia lyse AspA (Cj0087) catalyzing the deamination to fumarate (Guccione et al., 2008; Novik et al., 2010). This AspA-catalyzed reaction of aspartate to fumarate also plays a role in the response of *C. jejuni* 81–176 to high pressure and its recovery from cell injury (Bieche et al., 2012). Fumarate might be converted to oxaloacetate, which can be used as substrate for the gluconeogenesis and synthesis of essential carbohydrates. Alternatively, fumarate represents an important electron acceptor for the oxygen-independent respiration of *C. jejuni* (Sellars et al., 2002), generating a significant amount of succinate, which is secreted during the growth by *C. jejuni* (Guccione et al., 2008). In addition, aspartate is the precursor for the biosynthesis of several proteinogenic amino acids (lysine, methionine, threonine, isoleucine) as well as β-alanine. The latter is generated from aspartate by the activity of an aspartate alpha-decarboxylase PanD (Cj0296c) and serves as precursor for the synthesis of pantothenate and coenzyme A.

Genome sequence analysis showed that *C. jejuni* encodes for an asparaginase (AnsB) facilitating the conversion of asparagine to aspartate and for a glutamate synthase (GltBD) catalyzing the generation of glutamate from glutamine and 2-oxoglutarate (Gundogdu et al., 2007). These enzymes seem functional since asparaginase activity was detected in cell extracts of *C. jejuni* (Guccione et al., 2008) and ^14^C-labeled glutamine was catabolized under CO_2_ production (Westfall et al., 1986). These studies indicated that *C. jejuni* is able to catabolize asparagine as well as glutamine and both amino acids are chemoattractants for *C. jejuni* (Vegge et al., 2009). Although glutamine uptake in *C. jejuni* is facilitated by the conserved Paq transporter system (Lin et al., 2009), growth experiments revealed that just a subset of the tested *C. jejuni* isolates could use glutamine as sole carbon and energy source (Hofreuter et al., 2008). These results imply that the direct uptake of glutamine by the Paq and any additional transporter systems is not sufficient to promote pronounced proliferation of *C. jejuni* under the tested *in vitro* conditions. Only *C. jejuni* strains encoding for a secreted γ-glutamyltranspeptidase (GGT) were able to utilize glutamine efficiently for growth (Hofreuter et al., 2008). This periplasmic GGT enzyme facilitates the hydrolysis of glutamine to glutamate and ammonia similar to what has been described for *H. pylori* (Shibayama et al., 2007). Whereas the occurrence of GGT seems to be a conserved genetic trait in *H. pylori* (Leduc et al., 2010), not more than a third of the analyzed *C. jejuni* isolates harbored the GGT gene: Significant variations in the *ggt* frequency (between 8 and 30%) resulted from differences in the sequence type (ST) / clonal complex (CC) composition of the analyzed *C. jejuni* collections as determined by multilocus sequence typing (MLST): The *ggt* gene was strongly associated with ST-45 CC and ST-22 strains (Revez et al., 2011; de Haan et al., 2012) but typically absent in the common ST-21 isolates (Gripp et al., 2011; Zautner et al., 2011; de Haan et al., 2012). Surprisingly, GGT-activity provides *C. jejuni* strains not only an expanded amino catabolism but also a survival advantage in the presence of bactericidal isothiocyanates as shown for *C. jejuni* 81–176 (Dufour et al., 2012).

Most of the analyzed *C. jejuni* strains, including the reference strain NCTC 11168, were not able to grow with asparagine as the primary source of carbon and energy although all possess an L-asparaginase gene that encodes for a cytoplasmic AnsB enzyme (Hofreuter et al., 2008). This suggests that efficient asparagine import does not occur in *C. jejuni*. In contrast to *C. jejuni* NCTC 11168, certain isolates like *C. jejuni* 81–176 harbor an *ansB* allele variation that encodes for an additional sec-dependent secretion signal. Such a modified AnsB enzyme (AnsB^s^) is translocated to the periplasm where it catalyzes the deamination of asparagine to aspartate. Consequently, the growth of *ansB*^s^- and *ggt*-positive *C. jejuni* isolates with asparagine and glutamine relies on the conversion of both amino acids to aspartate and glutamate in the periplasm and the subsequent uptake of the deaminated amino acids (Hofreuter et al., 2008) through the DcuAB, DctA and Peb transporters (Leon-Kempis Mdel et al., 2006; Guccione et al., 2008). Interestingly, many *ggt*-positive *C. jejuni* strains encode also for a secreted asparaginase but lack the *fucP*-gene cluster, while *C. jejuni* isolates without the GGT- and AnsB^s^-mediated expanded amino acid catabolism harbor the genes required for fucose utilization (Gripp et al., 2011; Zautner et al., 2011; de Haan et al., 2012). This correlation may reflect the selection pressure to compensate the restricted amino acid catabolism with enhanced carbohydrate utilization. Future studies are required to elucidate how these differences in substrate utilization might correlate with a variable fine-tuning of the intermediary metabolism of GGT/AnsB^s^-positive and -negative *C. jejuni* strains and how these metabolic variations could reflect the physiological adaptations of certain *C. jejuni* strains to different hosts.

Besides the transport systems for the growth-promoting amino acids serine, proline, aspartate and glutamate, *C. jejuni* harbors homologs of the LIV (leucine, isoleucine, valine) branched-chain amino acid ABC transporter system of *E. coli* (Ribardo and Hendrixson, 2011). Though none of the branched-chain amino acids directly fuels the growth of *C. jejuni* (Guccione et al., 2008; Hofreuter et al., 2008), isoleucine interacts with the transducer-like protein Tlp3 of *C. jejuni* NCTC 11168 and induces a positive chemotactic response (Rahman et al., 2014). The LIV-locus in *C. jejuni* is comprised of six genes encoding for the periplasmic binding proteins LivJ and LivK, the two permeases LivH and LivM, and the cytoplasmic ATPases LivG and LivF. Targeted mutagenesis confirmed that the individual LIV ABC transporter components are responsible for the uptake of the branched amino acids, particularly of leucine (Ribardo and Hendrixson, 2011). Expression of the *liv* genes in *C. jejuni* is not repressed in the presence of leucine (Ribardo and Hendrixson, 2011) as shown for *E. coli* (Quay and Oxender, 1976), further exemplifying the metabolic specificity of *C. jejuni*.

Several additional putative amino acid transporters are present within the sequenced genomes of *C. jejuni*. For example, Cj0903c with homology to members of the sodium:alanine symporter family and SstT (Cj1097), the putative sodium/serine-threonine symporter, are conserved in *C. jejuni* and future studies are needed to elucidate their substrate specificity. Besides the aspartate/glutamate-binding protein Peb1A, *C. jejuni* harbors CjaA, CjaC and GlnH, three additional periplasmic proteins that belong to the family 3 of solute-binding proteins, which might also facilitate the uptake of amino acids. CjaA (Cj0982c) is an N-glycosylated 30 kDa lipoprotein attached to the inner membrane (Pawelec et al., 1997; Wyszynska et al., 2008) that binds cysteine (Müller et al., 2005). Its direct involvement in the import of cysteine has not been experimentally proven so far, and growth experiments suggested that CjaA might not be solely involved in the uptake of cysteine (Vorwerk et al., 2014). CjaC (Cj0734c) is a N-glycosylated 28 kDa protein with unknown substrate specificity that is anchored in the cytoplasmic membrane of *C. jejuni* and shows best homologies to various periplasmic binding proteins of ABC-type amino acid transporters (Pawelec et al., 1998; Wyszynska et al., 2007). Interestingly, a *hisJ* mutant of *S*. Typhimurium could be complemented by heterologous expression of the *C. jejuni cjaC* gene indicating that CjaC participates in the uptake of histidine in *C. jejuni* as well (Garvis et al., 1996). The third family 3 solute-binding protein, GlnH (Cj0817), remains uncharacterized. Taken together, though much progress has been made in recent years to characterize the amino acid catabolism of *C. jejuni*, future work is required to identify the transport systems involved in the uptake of amino acids that do not directly promote the growth of *C. jejuni* as energy or carbon sources.

Whereas the importance of amino acid uptake and utilization for the growth of *C. jejuni* is well documented, only few studies have investigated the *de novo* biosynthesis of amino acids in this pathogen, like e.g., the involvement of *ilvE* and *cysM* in the synthesis of leucine and cysteine, respectively (Ribardo and Hendrixson, 2011; Vorwerk et al., 2014). Other studies have examined the amino acid biosynthesis capability of *C. jejuni* by heterologous expression of certain genes of arginine (Hani and Chan, 1994; Hani et al., 1999), cysteine (Garvis et al., 1997), leucine (Labigne et al., 1992) and aromatic amino acid (Wösten et al., 1996) biosynthesis pathways in respective auxotrophic *Escherichia coli* mutants. Therefore, information about the complete biosynthesis pathways of amino acids are primarily obtained through the *in silico* analysis of available genome sequences of *C. jejuni* isolates: In contrast to *H. pylori*, which has limited capacity for the synthesis of amino acids and requires arginine, histidine, isoleucine, leucine, methionine, phenylalanine and valine for growth (Doig et al., 1999), *C. jejuni* NCTC 11168 encodes for enzymes enabling the synthesis of all amino acids (Gundogdu et al., 2007). Future studies are required to clarify how conserved the functionality of the predicted amino acid biosynthesis pathways are among *C. jejuni* strains as several amino acid auxotrophies, e.g., for methionine, proline or the branched amino acids isoleucine, leucine and valine, have been previously described in various *C. jejuni* isolates (Tenover et al., 1985; Blaser et al., 1986; Tenover and Patton, 1987).

### Peptidases and peptide catabolism of *C. jejuni*

The central role of amino acid catabolism for the proliferation of *C. jejuni* suggests that peptides may also be important growth-promoting substrates for this pathogen, especially, since the digestion of proteins in the gastrointestinal tract of its hosts generates a variety of peptides besides free amino acids (Adibi and Mercer, 1973). Several putative peptidases and proteases are encoded by the *C. jejuni* genome (Hofreuter et al., 2006; Gundogdu et al., 2007), and some, like ClpP (Cj0192c), HtrA (Cj1228c), CJJ81176_1086 (Cj1068), CJJ81176_1228 (Cj1215), Cj0511 or Pgp1 (Cj1345c; CJJ81176_1344), have been associated with the virulence of *C. jejuni* (Brondsted et al., 2005; Cohn et al., 2007; Novik et al., 2010; Boehm et al., 2012; Frirdich et al., 2012; Karlyshev et al., 2014).

The role of peptidases in the catabolism and nutrient acquisition of *C. jejuni* has not been characterized in detail so far. BIOLOG phenotype microarray analysis suggested that dipeptides like glycyl-glutamine and glycyl-proline enhance the respiratory activity of *C. jejuni* and can be used as carbon sources though strain specific differences exist (Gripp et al., 2011; Muraoka and Zhang, 2011). This variability in peptide catabolism might be the consequence of *C. jejuni* isolates having variable number of peptidases: GGT, the putative S15 family dipeptidyl-peptidase CJJ81176_1680 or the putative subtilisin-like serine peptidases Cj1365 / CJJ81176_1367 and CJJ81176_1371 occur in a subset of *C. jejuni* strains (Champion et al., 2005; Hofreuter et al., 2006; Hepworth et al., 2007; Gonzalez et al., 2009; Zautner et al., 2011). Only *ggt*-positive *C. jejuni* strains can efficiently use the tripeptide glutathione as carbon/energy (Hofreuter et al., 2008) and cysteine source (Vorwerk et al., 2014). A recent study using the BIOLOG phenotype microarray technology suggested that the *C. jejuni* NCTC 11168 transporter protein Cj0917c, which has homology to the carbon starvation protein A (CstA) of *E. coli*, is involved in the catabolism of several tri- and dipeptides (Rasmussen et al., 2013). Additional experiments are necessary to demonstrate the growth-promoting effect of respective peptides. Moreover, the *Campylobacter* peptide transporter A (CptA; CJJ81176_0236), a member of the Proton-dependent Oligopeptide Transporter (POT) family, has been described to promote the growth of *C. jejuni* 81–176 with the dipeptides Cys-Gly, Arg-Trp and Arg-Ile (Vorwerk et al., 2014).

Taken together, *C. jejuni* shows an intriguing metabolic diversity. The diverse growth properties of *C. jejuni* isolates result from the variable presence or absence of metabolic genes involved in the strain-specific utilization of particular substrates such as fucose, asparagine or glutamine and peptides. In addition, *C. jejuni* isolates are equipped with different sets of group A chemoreceptor *tlp* genes that response to a variety of potential nutrients (Day et al., 2012; Rahman et al., 2014). Such a variable presence of chemosensory receptor genes in *C. jejuni* suggests that different strains may not respond equivalently to certain nutrients and consequently cannot utilize and benefit from the same growth substrates.

## Finding the optimal oxygen level: how does a microaerophilic pathogen persists in the anaerobic environment of the intestine?

In contrast to facultative anaerobe pathogens like *S*. Typhimurium or enteropathogenic *E. coli*, *C. jejuni* faces the challenging situation to proliferate as obligate microaerophilic bacterium in the intestine of its hosts where lower levels than the preferred 5% oxygen exist. It has been suggested that *C. jejuni* is adapted to this disadvantageous circumstance by its colonization pattern and distinct respiratory capability: (i) *C. jejuni* colonizes preferentially the mucus layer and the intestinal crypt close to the epithelium (Lee et al., 1986; Beery et al., 1988) where the oxygen tension is higher than in the intestinal lumen. In addition, the region between the mid small intestine and the mid colon, which is the preferred colonization site of *C. jejuni*, harbors higher oxygen tensions than the distal colon and rectum (He et al., 1999). (ii) Early biochemical characterizations of *C. jejuni* demonstrated the presence of a surprisingly complex and highly branched respiratory chain, allowing this pathogen to use a variety of electron donors and several other electron acceptors besides oxygen (Carlone and Lascelles, 1982; Hoffman and Goodman, 1982; Hitchcock et al., 2010). Menaquinone-6 and methyl-substituted menaquinone-6 mediate in *C. jejuni* the electron transfer along the respiration chain between electron donors and receptors (Carlone and Anet, 1983; Moss et al., 1984). The *menBCDEF* menaquinone biosynthesis genes found in enterobacteria to catalyze the transformation of chorismate to menaquinone are absent in *C. jejuni*. Instead *C. jejuni* harbors enzymes of an alternative menaquinone biosynthesis pathway similar to the futalosine pathway described for *Streptomyces coelicolor* (Hiratsuka et al., 2008; Li et al., 2011). This modified futalosine pathway of *C. jejuni* is also employed by *H. pylori* (Arakawa et al., 2011) and uses 6-amino-6-deoxyfutalosine instead of futalosine as an intermediate for the synthesis of menaquinone. Consequently, orthologs to enzymes MqnA and MqnB of *S. coelicolor* are not required by *C. jejuni* and replaced by the menaquinone biosynthetic enzyme A2 (MqnA2; Cj1285c) and the 5′-methylthioadenosine nucleosidase MTAN (Cj0117) (Li et al., 2011). MTAN might be a promising new drug target for *C. jejuni* as demonstrated for *H. pylori* (Wang et al., 2012).

Genome sequence analysis of *C. jejuni* (Parkhill et al., 2000; Sellars et al., 2002) revealed that the respiratory electron chain of *C. jejuni* is comprised of two terminal, membrane-bound oxidases. Both enzymes mediate the oxygen-dependent respiration of *C. jejuni* but harbor strikingly different oxygen affinities: the cyanide-insensitive oxidase CioAB (initially named CydAB; Cj0081, Cj0082) exhibits low affinity for oxygen, whereas the cyanide-sensitive, *cb*-type cytochrome c oxidase CcoNOQP (Cj1490c-Cj1487c) shows high affinity to oxygen and might be crucial for respiration under oxygen-limited conditions (Jackson et al., 2007). The CioAB oxidase receives electrons directly from the oxidized menaquinone pool, whereas oxygen reduction by the CcoNOQP oxidase involves electron transfer from the oxidized menaquinone pool via the proton-translocating cytochrome *bc* complex (PetABC: Cj1186c-Cj1184c) and a periplasmic *c-type* cytochrome (Cj1153).

Though *C. jejuni* is equipped with various enzymes facilitating oxygen-independent respiration, no growth can be observed under strictly anaerobic conditions (Veron et al., 1981). It was suggested that the class I ribonucleotide reductase (NrdAB-type RNR) of *C. jejuni* is responsible for its inability to grow anaerobically because this enzyme requires low amounts of oxygen for the DNA synthesis (Sellars et al., 2002). This prerequisite for oxygen brings the disadvantage that non-specific electron transfer from the respiratory chain to oxygen occurs (Cabiscol et al., 2000), leading to the generation of toxic reactive oxygen species (ROS) like hydroxyl (^·^OH) and superoxide (O^−^_2_) radicals as well as hydrogen peroxide (H_2_O_2_). *C. jejuni* harbors a variety of ROS-detoxifying enzymes including the superoxide dismutase SodB (Pesci et al., 1994), the alkyl hydroxide reductase AhpC (Baillon et al., 1999), the catalase KatA (Cj1385) (Day et al., 2000) as well as the thiolperoxidases Tpx and Bcp (Atack et al., 2008). Surprisingly, these enzymes are unable to provide sufficient oxygen tolerance in aerobic conditions for *C. jejuni*. A recent study linked the oxygen-labile, iron-sulfur (4Fe-4S)-containing metabolic enzymes pyruvate:acceptor oxidoreductase POR and the *oorDABC* (*cj0535*-*cj0538*) encoded 2-oxoglutarate:acceptor oxidoreductase (OOR) to the oxygen sensitivity of *C. jejuni* (Kendall et al., 2014). The enzymatic activity of POR and OOR are partially protected by the hemerythrin proteins HerA (Cj0241c) and HerB (Cj1224), but *C. jejuni* lacks sufficient mechanisms to repair POR and OOR once damaged through exposure to atmospheric oxygen concentrations (Kendall et al., 2014).

### A variety of electron donors fuel the respiratory activity of *C. jejuni*

The reduction equivalents nicotinamide adenine dinucleotide (NADH) and flavin adenine dinucleotide (FADH) serve in many bacteria as major electron sources for the respiratory electron transport chain, which generates through the proton-translocating NADH:quinone oxidoreductase (Nuo/NDH-1) complex a proton gradient that drives the oxidative phosphorylation (Haddock and Jones, 1977). Strikingly, NADH is a poor respiratory electron donor in *C. jejuni* in contrast to FADH (Hoffman and Goodman, 1982) as consequence of the specific NDH-1 complex property encoded by the *nuo* gene cluster *cj1566c* to *cj1579c* in *C. jejuni* NCTC 11168: The genes *nuoE* and *nuoF* encoding for the NADH dehydrogenase subunits of the NDH-1 complex are replaced in *C. jejuni* NCTC 11168 by the genes *cj1575c* and *cj1574c* (Smith et al., 2000). It was shown that Cj1574c, probably in conjunction with Cj1575c, mediates the electron transfer from the reduced, flavin monoculeotide containing flavodoxin FldA (Cj1382c) to the NDH-1 complex (Weerakoon and Olson, 2008). Consequently, the NDH-1 complex of *C. jejuni* seems to participate rather in the oxidation of flavin mononucleotides than of NADH. It was further demonstrated that reduced FldA is generated by the oxidation of 2-oxoglutarate to succinyl-CoA catalyzed by the OOR, whereas the putative ferredoxins FdxA (Cj0333c) and Cj0369c are no electron acceptors for OOR (Weerakoon and Olson, 2008).

Several other electron donors besides FADH have been described to fuel the respiration chain of *C. jejuni*: some of them, like hydrogen, formate, lactate or succinate, are generated through the catabolic activity of the host gut microbiota (Bernalier-Donadille, 2010), which suggests that *C. jejuni* might benefit from metabolic cross-feeding. Hydrogen can be used as an electron donor by *C. jejuni* through the activity of the membrane-bound NiFe-type hydrogenase HydABCD (Cj1267c-Cj1264c) (Hoffman and Goodman, 1982; Weerakoon et al., 2009). The accessory factors encoded by the *hypFBCDEA* operon (*cj0622-cj0627*) are required for assembling of the hydrogenase enzyme complex and insertion of the nickel cofactor. An ABC-transporter system (Cj1584c-Cj1580c) of *C. jejuni* NCTC 11168 has recently been identified as a high-affinity nickel uptake system and was named NikZYXWV (Howlett et al., 2012). Under low nickel concentrations the inactivation of *nikZ* (*cj1584c*), encoding for a periplasmic binding protein, led to an abolished hydrogenase activity of the mutant strain. This result demonstrated the importance of the Nik-transporter system for the acquisition of nickel as a cofactor for the enzyme. Yet hydrogenase activity of the *nik*Z mutant was observed in the presence of high nickel concentrations, indicating the presence of additional nickel transporters (Howlett et al., 2012). In contrast to studies with *E. coli*, showing the importance of the nickel chaperone SlyD for the hydrogenase activity, a mutation in the *slyD* ortholog gene *cj0115* of *C. jejuni* NCTC 11168 did not abolish the mutants nickel uptake capacity and hydrogenase activity (Howlett et al., 2012).

Formate is mainly generated by the mixed-acid fermentation of the intestinal microbiota and is sensed as chemoattractant by *C. jejuni* through the Tlp7 chemoreceptor (Vegge et al., 2009; Tareen et al., 2010). It can be oxidized by *C. jejuni* to CO_2_, protons and electrons through a membrane-bound formate dehydrogenase (FDH) complex comprised of the selenocysteine-containing subunit FdhH (Cj1511c), the iron-sulfur subunit FdhB (Cj1510c) and the formate dehydrogenase cytochrome-b subunit FdhC (Cj1509c), which requires FdhD (Cj1508c) for its activity. The released electrons are directly transferred from the FDH complex to the menaquinone pool (Weerakoon et al., 2009). The FDH activity of *C. jejuni* is controlled by the accessory proteins FdhT (Cj1500), FdhU (Cj1501) and a high-affinity TupABC-like tungstate transporter (Cj1538-Cj1540) indicating that tungstate might be incorporated into the FDH complex (Smart et al., 2009; Pryjma et al., 2012; Shaw et al., 2012).

Lactate is oxidized by *C. jejuni* to pyruvate by the membrane-associated NAD-independent respiratory lactate dehydrogenase complex (L-iLDH; Cj0075c, Cj0074c; Cj0073c) though inactivation of these genes in *C. jejuni* NCTC 11168 did not abolish the growth of respective mutants with lactate (Thomas et al., 2011). However, a second L-iLDH, the oxidoreductase Cj1585c, was identified and demonstrated to be responsible for the observed redundancy in the catabolism of L-lactate. Only a double mutation inactivating *cj0075c* and *cj1585c* abolished the growth of *C. jejuni* NCTC 11168 with 20 mM L-lactate as a carbon source but it did not affect in the utilization of D-lactate. The gene locus of *cj1585c* is not conserved in *C. jejuni* (Hofreuter et al., 2006) and is replaced by a dimethyl sulfoxide reductase (*dmsABC*) gene cluster encoding for an anaerobic dimethyl sulfoxide reductase complex in strains like *C. jejuni* 81–176 (Hofreuter et al., 2006), 81116 (Pearson et al., 2007), M1 (Friis et al., 2010) or 327 (Takamiya et al., 2011). This finding is verified by the observation that a mutant in the Cj0075c homolog of wild-type strain *C. jejuni* 81116, which naturally lacks the L-iLDH Cj1585c, was unable to grow with L-lactate (Thomas et al., 2011).

The TCA cycle intermediate succinate is not only a carbon and energy source for *C. jejuni* but serves also as an electron donor. Consequently, succinate is oxidized to fumarate accompanied with the generation of FADH_2_ and the subsequent electron transfer to the menaquinone pool. One predicted succinate dehydrogenase (succinate:quinone oxidoreductase) SdhABC (Cj0437-Cj0439) of *C. jejuni* was misannotated and not involved in the conversion of succinate to fumarate (Weingarten et al., 2009). Instead, the putative FrdABC fumarate reductase complex comprised of a membrane-associated diheme cytochrome B (FrdC, Cj0408), the flavoprotein FrdA (Cj0409) and the Fe-S protein FrdB (Cj0410) showed properties of a succinate:quinone reductase and was solely responsible for the oxidation of succinate to fumarate (Weingarten et al., 2009).

*C. jejuni* is unable to catabolize gluconate due to the absence of an Entner-Doudoroff pathway, but it can use gluconate as electron donor through a temperature-regulated flavin-containing gluconate dehydrogenase (Pajaniappan et al., 2008). Proteomic analysis demonstrated that the gluconate dehydrogenase (GADH) expression increased in *C. jejuni* upon a temperature shift from 37 to 42°C correlating with an elevated GADH activity. The co-transcribed genes *cj0414* and *cj0415* encode for the two components of the gluconate-oxidizing oxidoreductase, which is predicted to be localized in the periplasm peripherally associated with the cytoplasmic membrane and to transfer electrons to the periplasmic cytochrome *c* (Pajaniappan et al., 2008). Interestingly, both GADH subunits are conserved in *C. jejuni* but absent from other *Campylobacter* species.

Another example of the unique respiratory capacity of *C. jejuni* is its sulfite respiration system, which uses sulfite and metabisulfite as electron donors. This sulfite:cytochrome c oxidoreductase (SOR) system is conserved in *C. jejuni* and can also be found in *C. lari* but not in any other examined *Campylobacter* and *Helicobacter* species. The periplasmic proteins SorA (Cj0005c) and SorB (Cj0004c) with properties of a molybdopterin oxidoreductase and a monoheme cytochrome c_552_, respectively, catalyze the oxidation of sulfite to sulfate accompanied by an electron transfer to cytochrome *c* (Myers and Kelly, 2005).

In summary, the highly branched electron transport chain of *C. jejuni* enables respiration with a variety of electron donors that are secreted catabolic end products of the surrounding microbiota of the intestinal habitat. This cross-feeding might provide an important advantage for *C. jejuni* to overcome the colonization resistance of its host and to establish its nutritional niche.

### The versatility of oxygen-independent respiration in *C. jejuni*

*C. jejuni* is unable to grow under anaerobic conditions but is well adapted to the oxygen-limited conditions of its intestinal habitat by harboring a variety of respiration systems using alternative electron acceptors to oxygen, like nitrate, nitrite, S- and N-oxides or fumarate (Sellars et al., 2002).

Under oxygen-limited cultivation conditions of *C. jejuni*, fumarate is reduced to succinate by the bifunctional FrdABC complex and the methlymenaquinol:fumarate reductase complex comprised of MrfA (Cj0437), MrfB (Cj0438) and MrfE (Cj0439), initially described as SdhABC complex (Weingarten et al., 2009; Guccione et al., 2010). While the fumarate reductase FrdABC complex acts in the cytoplasm of *C. jejuni*, the MfrABE-mediated fumarate reduction occurs in the periplasm where MfrA is exported by the twin arginine translocase (TAT) system (Hitchcock et al., 2010). The periplasmic MrfABE complex of *C. jejuni* also participates in the reduction of the C4-dicarboxylate mesaconate and the C4-monocarboxylate crotonate (Guccione et al., 2010). Interestingly, both substances are fumarate analogs, which are produced as metabolic intermediates by anaerobic bacteria of the intestinal microbiota, suggesting a metabolic interaction between *C. jejuni* and species of *Clostridium* and *Fusobacterium* (Guccione et al., 2010).

Dimethyl sulfoxide (DMSO) and trimethylamine *N*-oxide (TMAO) are additional electron acceptors of *C. jejuni in vitro* (Sellars et al., 2002): The gene *cj0264c* of *C. jejuni* NCTC 11168 is conserved in *C. jejuni* and encodes for a molybdopterin-containing oxidoreductase, which is responsible for its DMSO and TMAO reductase activity. The TMAO- and DMSO-dependent respiration improved the *in vitro* growth of *C. jejuni* NCTC 11168 under oxygen-limited but not under microaerobic conditions. Future studies have to clarify if the putative cytochrome C-type heme-binding periplasmic protein, encoded by *cj0265c*, participates in the elector transfer to DMSO, TMAO and other *N*- or *S*- oxides (Sellars et al., 2002). Beside Cj0264c, *C. jejuni* 81–176 harbors a putative oxidoreductase with homology to the anaerobic dimethylsulfoxide reductase DmsA (Hofreuter et al., 2006). This oxidoreductase has 28% protein sequence identity with Cj0264c and may catalyze the reduction of DMSO / TMAO as well. The *dmsA* gene (*cju34* / *cjj81176_1570*) of *C. jejuni* 81–176 is organized in a gene cluster together with three other gene genes encoding for the iron-sulfur containing DMSO reductase subunit DmsB (CJJ81176_1571), the DMSO reductase anchor subunit DmsC (CJJ81176_1572) and chaperon protein TorD (CJJ81176_1572) that are predicted to participate with DmsA at the electron transfer to DMSO / TMAO or other S- and N-oxides (Hofreuter et al., 2006).

Several human pathogens harbor nitrate and nitrite respiration systems (Sparacino-Watkins et al., 2014). *C. jejuni* encodes for the periplasmic located Nap-type nitrate reductase (Cj0780-Cj0785) catalyzing the reduction of nitrate to nitrite (Pittman and Kelly, 2005). Nitrite can subsequently be used as electron acceptor and reduced to ammonia by *C. jejuni* through the concerted activity of the pentaheme nitrite reductase NrfA (Cj1357c) and the electron donor protein NrfH (Cj1358c) of the tetraheme NapC/NirT cytochrome C family (Pittman et al., 2007). Exposure to nitrite leads to a nitrosative stress response in *C. jejuni* and the nitrite reductase NrfA catalyzes not only the reduction of nitrite to ammonia but participates with the single domain globin Cgb (Cj1586) in the detoxification of nitric oxide (Pittman et al., 2007).

*S*. Typhimurium uses the TtrRSBCA system for the oxygen-independent respiration with the alternative electron acceptor tetrathionate (Hensel et al., 1999). The recently described thiosulfate/tetrathionate-dependent respiration of *C. jejuni* is mediated by the bifunctional tetrathionate reductase/thiosulfate dehydrogenase TsdA (Liu et al., 2013) and seems to be a variable property in *C. jejuni* that promotes growth only of certain isolates in oxygen-limited conditions. Some *C. jejuni* strains like 81116 (Pearson et al., 2007) or M1 (Friis et al., 2010) harbor an active TsdA (C8j_0815) enzyme that catalyzes the reduction of tetrathionate to thiosulfate in the presence of additional electron donors like formate (Liu et al., 2013). In addition, these strains can use thiosulfate as an electron donor that is converted by TsdA to tetrathionate under microaerobic conditions. The *C. jejuni* isolates NCTC 11168 (Parkhill et al., 2000), RM1221 (Fouts et al., 2005) or 81–176 (Hofreuter et al., 2006) harbor truncated alleles of *tsdA* (*c8j_0815*) but possess like *C. jejuni* 81116 a less-active TsdA homolog (C8j_0040), which catalyzes the conversions between thiosulfate and tetrathionate at low levels (Liu et al., 2013). Future studies are needed to examine if the minimal thiosulfate oxidation and tetrathionate reduction driven by C8j_0040 can contribute to the growth of the *C. jejuni* strains harboring truncated *tsdA* genes. Furthermore, animal infection studies are required to clarify if tetrathionate respiration in *C. jejuni* provides a similar benefit for the colonization process as described for *S*. Typhimurium (Winter et al., 2010).

## Physiological factors promoting the colonization process of *C. jejuni*

Transposon mutagenesis projects and *in silico* analysis of metabolic pathways led to the identification of genes essential for the proliferation of *C. jejuni in vitro* (Metris et al., 2011; Stahl and Stintzi, 2011). In addition, recent studies provided comprehensive data of genes expressed during the *in vitro* growth of *C. jejuni* when cultivated in nutrient rich media like BHI or Brucella broth by high-throughput deep sequencing of messenger RNA transcripts (Chaudhuri et al., 2011; Dugar et al., 2013; Porcelli et al., 2013). However, the knowledge of the nutritional requirements and metabolic traits that facilitate the growth of *C. jejuni* during the colonization of various hosts is limited. Still, our understanding about the metabolic necessities of *C. jejuni* required for a successful host infection has significantly improved in recent years by *in vivo* transcriptome analyses and screening of isogenic *C. jejuni* mutants in colonization models using 1 day old chickens, neonatal piglets and immunodeficient, antibiotic treated or germfree mice.

**Figure 1:**
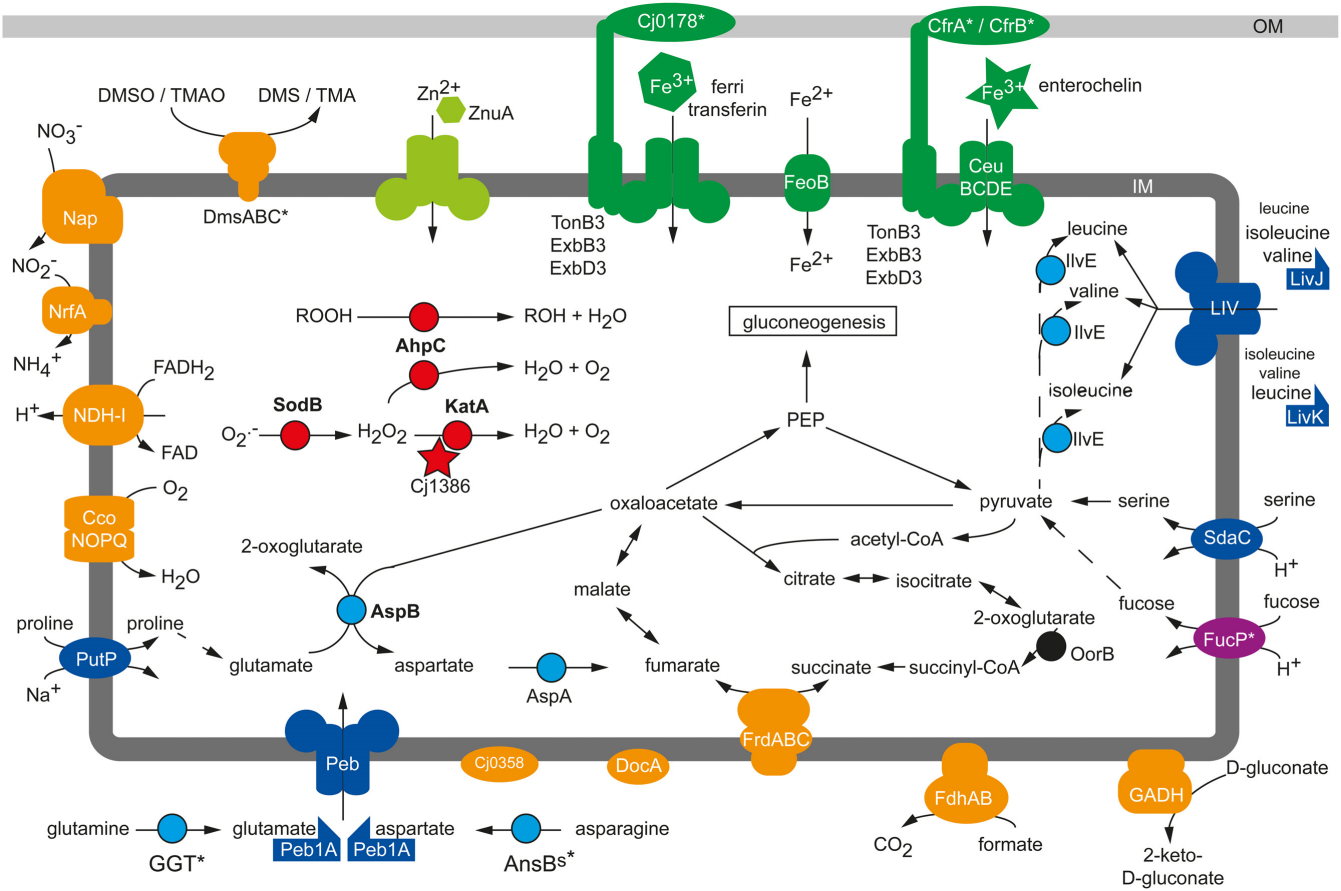
**Physiological colonization factors of *C. jejuni***. Schematic presentation of metabolic factors being required for the efficient colonization process of *C. jejuni* in chicken, murine and porcine infection experiments. Blue, amino acid metabolism; red, reactive oxygen defense; orange, respiration; green, micronutrient utilization; IM, inner membrane; OM, outer membrane, *, Strain-specific gene; not conserved in all *C. jejuni* isolates.

Taveirne et al. (2013) applied the RNAseq approach to study the *in vivo* transcriptome of *C. jejuni* after infection of 1-day-old chicks. Comparing the gene expression profile of *C. jejuni* isolated from the caecum of the chicks with the transcriptome during *in vitro* cultivation identified several gene groups that were more highly expressed *in vivo* including, for example, genes involved in the oxidative stress response (*katA*), iron (*chuABCD*; *cjj81176_1601*-*cjj81176_1604*) and phosphate (*pstSCAB*; *cjj81176_0642*-*cjj81176_0645*) transport. Elevated expression of the single-domain globin gene *cbg* (Elvers et al., 2004) indicated that *C. jejuni* was also exposed to nitrosative stress during colonization of the chicken caecum (Taveirne et al., 2013). One of the most increased transcripts *in vivo* was *katA* (Taveirne et al., 2013), the product of which is essential to counteract the damaging effect of H_2_O_2_(Grant and Park, 1995). Such oxidative stress might occur through the production of H_2_O_2_ by the intestinal epithelial cells at the colonization site of *C. jejuni* as indicated by *ex vivo* experiments with biopsy samples and infection of cultured HCT-8 cells (Corcionivoschi et al., 2012). In addition, the production of reactive oxygen species like superoxide anions and H_2_O_2_ are central for the innate immune defense (Rada and Leto, 2008). The KatA-mediated detoxification of H_2_O_2_ is crucial for *C. jejuni in vivo*, as the inactivation of *katA* reduced the colonization of the chicken caecum by the respective *C. jejuni* mutant (Bingham-Ramos and Hendrixson, 2008; Palyada et al., 2009). The severity of the observed colonization defects differed between these two studies, and this variability is likely due to dissimilar experimental settings or different *C. jejuni* and chicken strains used in the experiments. The ankyrin repeat-containing protein Cj1386 of *C. jejuni* NCTC 11168 is required for its full catalase activity by facilitating the correct heme trafficking to KatA (Flint et al., 2012). Similar to the *katA* mutant, a *cj1386* mutant exhibited a reduced capability to colonize the caecum of chicken and to compete with the wild-type strain in the intestinal colonization of neonate piglets (Flint et al., 2012), further illustrating the importance of H_2_O_2_ detoxification for the pathogenesis of *C. jejuni* (Figure 1). The periplasmic c-type cytochrome Cj1153 has been suggested to participate in the defense against H_2_O_2_through the reduction and detoxification of exogenous H_2_O_2_to water and oxygen via electron transfer to the potential *cytC* peroxidases DocA (Cj0020c) and Cj0358 (Sellars et al., 2002; Hendrixson and DiRita, 2004). But in contrast to KatA, both peroxidases play no role in the resistance of *C. jejuni* against H_2_O_2_; nonetheless *docA* and *cj0358* mutants were defective in the colonization of chicken (Hendrixson and DiRita, 2004; Bingham-Ramos and Hendrixson, 2008). The up-regulation of *katA* and additional oxidative stress response genes like *sodB*, *ahpC* and *tpx* was also observed in a microarray study analyzing the gene expression profile of *C. jejuni* in a rabbit ileal loop infection model (Stintzi et al., 2005). This observation hinted once more to the importance to counteract oxidative stress for a successful colonization process of *C. jejuni* and was confirmed by infection experiments demonstrating that AhpC and SodB activities promote the colonization of *C. jejuni* in chickens and mice (Palyada et al., 2009; Novik et al., 2010).

Microarray-based gene expression analysis of *C. jejuni* isolated from the caecum of infected chickens suggested that an optimal respiratory adaptation of *C. jejuni* to its intestinal habitat is central for the colonization process (Woodall et al., 2005). This is reflected by the upregulation of the *petABC* and *ccoNOQP* operons *in vivo*, both of which facilitate the enhanced respiration of *C. jejuni* with the *cb*-type cytochrome c oxidase, which harbors high affinity to oxygen. Mutation of *ccoNOQP* abolished the ability of *C. jejuni* to colonize chicken, illustrating the crucial role of the terminal oxidoreductase for this microaerophilic pathogen to maintain its oxygen-dependent respiratory activity in the avian gastrointestinal tract (Weingarten et al., 2008). In contrast, inactivation of the terminal cyanide-insensitive oxidase CioA did not affect the colonization process (Weingarten et al., 2008), though the *cioA* expression was significantly higher *in vivo* than *in vitro* (Stintzi et al., 2005). Infection experiments with isogenic mutants of the highly branched respiratory chain revealed that especially the usage of alternative electron acceptors is crucial for the persistence of *C. jejuni* in the oxygen-limited environment of the intestine (Figure 1): The *nap* and *nrf* genes, facilitating the oxygen-independent respiration with nitrate and nitrite, were up-regulated *in vivo* (Woodall et al., 2005) and the NapA-mediated nitrate respiration of *C. jejuni* supported colonization in chickens (Weingarten et al., 2008). Interestingly, an increased intestinal nitrate level produced through an inflammatory host response also promoted the anaerobic respiration and luminal growth of *E. coli* in a murine infection model (Winter et al., 2013). Though the nitrite reductase activity of *C. jejuni* participates in nitrite respiration and protects against nitrosative stress *in vitro*, a *nrfA* mutant showed no general chicken colonization defect of the respective mutant in two independent studies (Pittman et al., 2007; Weingarten et al., 2008). Such discrepancy might be the result of different *C. jejuni* and chicken strains used in the studies as well as dissimilar infection doses and protocols. DMSO and TMAO have been proposed as electron acceptors that could be used by *C. jejuni* in aquatic environments since neither substances is found in significant amounts in the gastrointestinal tract of animals (Sellars et al., 2002). However, TMAO might eventually be present in the gastrointestinal tract under certain circumstances, as its precursor TMA is commonly produced by the human microbiota through the catabolism of carnitine and oxidized to TMAO in the host liver (Koeth et al., 2013; Zhu et al., 2014). This could explain why the inactivation of the *dmsA* gene in *C. jejuni* 81–176 resulted in a mutant with diminished persistence capability in immunodeficient mice when co-infected with the wild-type strain (Hofreuter et al., 2006). In contrast, the TMAO/DMSO reductase Cj0264c of *C. jejuni* NCTC 11168 was in single-infection experiments negligible for the colonization of chicken (Weingarten et al., 2008). Further work is needed to clarify if these different observations are the consequences of co- vs. single-infections, distinctive functions of DmsA and Cj0264c, or result from the dissimilarities in the *C. jejuni* strains and animal infection models used. Additional studies are required to identify the definite electron acceptors for the DmsA and Cj0264c oxidoreductases during the persistence of *C. jejuni* in the host intestine.

Increased *in vivo* gene expression levels were found for the C4-dicarboxylate transporter genes *ducA* and *dcuB*, the aspartase gene *aspA*, the fumarate reductase *frdABC* genes and the methylmenaquinol:fumarate reductase *mfr* genes (Woodall et al., 2005) participating in the fumarate respiration of *C. jejuni* under microaerobic and oxygen-limited conditions (Guccione et al., 2008, 2010). Higher *in vitro* expression of these genes was also observed under microaerobic and oxygen-limited growth conditions for the robust colonizer *C. jejuni* NCTC11168-O in comparison to its poor-colonizing variant *C. jejuni* NCTC11168-GS, likewise hinting toward an important role of oxygen-independent respiration for the persistence of *C. jejuni* in its host (Gaynor et al., 2004). Unexpectedly, the MrfA activity was dispensable for the *C. jejuni* chicken colonization (Weingarten et al., 2008), besides the impaired fumarate respiration under oxygen-limited conditions (Guccione et al., 2010). In contrast, the bifunctional fumarate reductase FrdA was required for optimal colonization (Weingarten et al., 2009). It is not yet clear to what extent the abolished utilization of growth substrates like the TCA cycle intermediates succinate or 2-oxoglutarate is responsible for the colonization defect of the *frdA* mutant in comparison to its inability to perform fumarate respiration. The importance of a functional TCA cycle for a *C. jejuni* infection was shown by the inactivation of the 2-oxoglutarate:acceptor oxidoreductase (*oorB*) resulting in a mutant with significantly reduced colonization capability in chicken compared to the wild-type strain as well (Weerakoon et al., 2009).

Intestinal pathogens have to overcome the gut microbiota-mediated colonization resistance of the host in order to establish a stable infection (Stecher et al., 2013). This might involve the successful competition with the intestinal microflora for common nutrients or, more often, the occupation of a specific metabolic niche in order to circumvent a direct competition with the commensal gut bacteria (Kamada et al., 2013). Murine infection models demonstrated that microbiota-derived molecular hydrogen promotes the growth of *H. pylori* (Olson and Maier, 2002) and *S*. Typhimurium (Maier et al., 2013) in a hydrogenase dependent manner, but hydrogenase activity of *C. jejuni* was not required for the persistence in chicken (Weerakoon et al., 2009). Other defects in systems fueling the respiration chain with electrons, like mutation in the formate dehydrogenase Fdh and the NDH-I complex, reduced the colonization capability of *C. jejuni* in chicken (Weerakoon et al., 2009). The gluconate dehydrogenase GADH, mainly active at 42°C, provided a host-specific benefit and was required by *C. jejuni* to persist in the chicken but not in the murine intestine (Pajaniappan et al., 2008), probably reflecting the different body temperatures of the avian and murine host. Whereas *C. jejuni* has to compete with commensal *E. coli* for gluconate (Chang et al., 2004), it generally does not catabolize carbohydrates that are utilized by commensal *E. coli*. *C. jejuni* strains harboring the FucP transporter are able to catabolize fucose (Muraoka and Zhang, 2011; Stahl et al., 2011), which is one of the less preferred growth substrates used by commensal and pathogenic *E. coli in vivo* (Fabich et al., 2008). The utilization of fucose is not essential for *C. jejuni in vivo*, as *fucP* mutants could be recovered from mono-infected chickens in similar amounts as the wild-type and had no disadvantage in chicken co-infection experiments with high infectious doses (Muraoka and Zhang, 2011). In low dose co-infection experiments with chicken the wild-type strain either outcompeted the *fucP* mutant (Muraoka and Zhang, 2011) or had no advantage (Stahl et al., 2011). In latter study, the additional administration of fucose allowed the wild-type strain to outcompete the mutant (Stahl et al., 2011), suggesting that sufficient amount of free fucose is not always available for *C. jejuni* under normal circumstances. Still, fucose catabolism seems to support the colonization of *fucP*-positive *C. jejuni* isolates in certain settings since a *C. jejuni fucP* mutant was recovered in lower numbers than the wild-type strain from co-infection experiments with neonatal piglets (Stahl et al., 2011).

The catabolism of free amino acids, derived through proteolytic protein degradation by the host and its intestinal microbiota, plays a central role for the colonization process of *C. jejuni* (Figure 1). Microarray experiments showed the upregulation of the serine dehydratase *sdaA* gene in *C. jejuni* colonizing the chicken caecum (Woodall et al., 2005), and an *sdaA* mutant with abolished serine utilization exhibited a severe colonization defect in chicken (Velayudhan et al., 2004) and mice (Hofreuter et al., 2012). Similarly, the GGT-dependent utilization of glutamine/glutathione contributed to the colonization of *C. jejuni* in the murine and avian intestine (Hofreuter et al., 2006; Barnes et al., 2007). Furthermore, a *C. jejuni peb1A* mutant, abolished in the growth with glutamate and aspartate *in vitro*, showed a colonization defect in mice, as did a *putP* mutant unable to catabolize proline (Pei et al., 1998; Hofreuter et al., 2012). With the exception of serine, the usage of amino acids provided no general but rather a tissue-specific benefit for *C. jejuni* during infection experiments with immune deficient Myd88^−^/^−^ mice: Glutamine / glutathione and proline catabolism were required for the efficient colonization of the murine intestine, but had no supporting effect during the persistence of *C. jejuni* in the murine liver after intraperitoneal infection (Hofreuter et al., 2006, 2008, 2012). In contrast, asparagine utilization through the secreted asparaginase AnsB^*s*^ did not promote the intestinal colonization of *C. jejuni* 81–176 but rather enhanced its colonization in the liver of mice (Hofreuter et al., 2008). Future studies have to clarify if the observed colonization defect of the *C. jejuni ansB*^s^ mutant in the murine liver is solely the consequence of a growth disadvantage or if the secreted asparaginase of *C. jejuni* has an additional immune modulatory effect as described for *S*. Typhimurium (Kullas et al., 2012). The asparaginase-catalyzed deamination of asparagine leads to aspartate, which represents a growth substrate but also a precursor of the alternative electron acceptor fumarate for *C. jejuni*, as describe above. Thus, the colonization defect in chicken and mice of an *aspA* mutant (Guccione et al., 2008; Novik et al., 2010) might be the combined effect of a reduced carbon and energy source availability as well as a diminished capability to perform fumarate respiration in the oxygen-limited intestinal environment. Moreover, the *C. jejuni* aspartate aminotransferase *aspB* mutant was defective in mouse colonization (Novik et al., 2010). Amino acids that cannot be used as energy sources by *C. jejuni* promote its infection process as well. This observation was made in chicken infection experiments with a *C. jejuni* transposon mutant library that identified the putative amino acid transporter Cj0903c and the periplasmic binding protein LivJ of the LIV branched-chain amino acid ABC transporter as colonization factors of *C. jejuni* (Hendrixson and DiRita, 2004). Additionally, inactivation of the periplasmic binding proteins LivK, which facilitates primarily the high level uptake of leucine, showed a reduced colonization capacity of *C. jejuni* in chicken when a low inoculum dose was used (Ribardo and Hendrixson, 2011). The mutation of the branched-chain amino acid aminotransferase *ilvE* gene resulted in a similar colonization phenotype as observed for the *livJ* and *livK* mutants. Unexpectedly, inactivation of other components of the LIV ABC transporter system did not affect the colonization levels of *C. jejuni* in chicken, indicating that other permeases might be involved in the uptake of the branched amino acids as well (Ribardo and Hendrixson, 2011).

*C. jejuni* requires not only energy sources but also micronutrients like iron, nickel, molybdate, tungsten, cobalt and zinc (Stahl et al., 2012) for the successful competition with the host microbiota. The growth-supporting effect of zinc has been demonstrated *in vivo* (Figure 1): A *C. jejuni* mutant with an inactivated periplasmic binding protein ZnuA (Cj0143c) of a high-affinity zinc ABC transporter system (Cj0143c-Cj0141c) showed a caecum colonization defect in chickens with normal microbiota (Davis et al., 2009) but not in chickens harboring a limited intestinal flora (Gielda and DiRita, 2012). Interestingly, an increased amount of zinc, magnesium and iron was measured in the caecum of chickens with limited flora whereas the concentrations of copper and manganese were reduced (Gielda and DiRita, 2012).

In addition to zinc, the availability of iron plays a central role in the pathogenesis of pathogens (Braun, 2001). Iron occurs in the host organism generally in its oxidized form as ferric iron (Fe^3+^). To reduce the availability of Fe^3+^ for pathogens, specific iron-binding proteins like lactoferrin and transferrin sequester ferric iron in host organisms. To overcome this iron limitation commensal and pathogenic bacteria secret siderophores, specific iron chelators with higher affinity to Fe^3+^ than the iron-binding proteins of the host (Skaar, 2010). Several studies have demonstrated that the acquisition of iron is important for its infection process. However, *C. jejuni* does not produce and encode Fe^3+^-binding siderophores (Baig et al., 1986; Gundogdu et al., 2007). Instead, it benefits from the host and its microbiota by encoding for several iron uptake systems that bind and import the iron-chelating siderophores of other bacteria such as ferric-enterobactin and salmochelin, the fungal ferrichrome or the host-derived iron-binding substances transferrin/lactoferrin, hemin and hemoglobin (Miller et al., 2009; Naikare et al., 2013). The outer membrane ferric-enterobactin receptor CfrA (Cj0755) of *C. jejuni* NCTC 11168 is expressed under iron-restricted growth conditions and required for colonization of chicken (Palyada et al., 2004). Interestingly, the robust colonizer *C. jejuni* 81–176 lacks the *cfrA* gene (Hofreuter et al., 2006), as do various other *C. jejuni* strains (Zeng et al., 2013a). These strains harbor the alternative ferric-enterobactin receptor *cfrB* (*cjj81176_0471*), which occurs in *C. jejuni* NCTC 11168 and other *cfrA*-positive strains as a pseudogene (Xu et al., 2010). Inactivation of *cfrB* in the bovine *C. jejuni* isolate JL11, which lacks the *cfrA* gene, clearly demonstrated that CfrB is required by this strain for the colonization of chicken (Xu et al., 2010). The CeuBCDE (Cj1352-Cj1355) ABC transporter system mediates, in cooperation with CfrA, the uptake of enterobactin/enterochelin into *C. jejuni*, and a *C. jejuni* NCTC 11168 *ceuE* mutant showed a similar chicken colonization defect as the *cfrA* mutant (Palyada et al., 2004). A further putative siderophore receptor is encoded by *cj0178* in *C. jejuni* NCTC 11168 and RM1221 but is absent in the isolate *C. jejuni* 81–176 (Hofreuter et al., 2006). The outer membrane protein Cj0178 (CtuA) was characterized as a receptor for the iron-binding host glycoproteins ferri-transferrin, ferri-lactoferrin and ferri-ovotransferrin (Miller et al., 2008). A *cj0178* mutant strain of *C. jejuni* NCTC 11168 showed a severe colonization defect in chicken infection experiments (Palyada et al., 2004) and a slightly attenuated phenotype in the rabbit ileal loop model (Stintzi et al., 2005). The *chu*ABCDZ (*cj1613c*-*cj1617*) gene cluster is widespread in *C. jejuni* and encodes for an iron uptake system that facilitates the utilization of the host compounds like hemoglobin and hemin as iron sources (Pickett et al., 1992; Ridley et al., 2006). Microarray and RNAseq analysis revealed the upregulation of *chu* genes in *C. jejuni* NCTC 11168 and 81–176 isolated from the caecum of infected chicken (Woodall et al., 2005; Taveirne et al., 2013), suggesting that the utilization of the host-derived heme is required for the colonization process of *C. jejuni*. However, a *C. jejuni* NCTC 11168 *chuA* mutant showed the same ability to colonize the chicken intestine as the wild-type strain (Naikare et al., 2013).

The uptake of bacterial or host-derived siderophores by *C. jejuni* requires, in addition to specific receptor and ABC transporter proteins, a periplasma-bridging TonB-ExbB-ExbD system, which energizes the translocation of the siderophores across the outer membrane (Braun, 2001). *C. jejuni* isolates are equipped with different numbers of TonB-ExbBD systems: While *C. jejuni* NCTC 11168 encodes for three TonB-ExbB-ExbD systems, *C. jejuni* 81–176 harbors only the TonB2-ExbB2-ExbD2 system, which seems to be conserved in *C. jejuni* (Hofreuter et al., 2006; Zeng et al., 2013b). It was recently demonstrated that TonB3 of *C. jejuni* NCTC 11168 mediates the uptake of a wide range of siderophores from bacteria and vertebrates, including enterobactin and salmochelin, and a *tonB3* mutant showed an abolished colonization capability in chicken infection experiments (Naikare et al., 2013). Moreover, a *C. jejuni* NCTC 11168 *tonB1* mutant exhibited like the *tonB3* mutant, an attenuated colonization phenotype in chicken, whereas the inactivation of *tonB2* in *C. jejuni* NCTC 11168 did not dramatically affect the enterobactin utilization as well as colonization capability (Naikare et al., 2013). *C. jejuni* relies not only on the utilization of siderophore-bound ferric iron but can also use ferrous (Fe^2+^) iron. Some *C. jejuni* strains take up Fe^2+^ through the FeoB transport protein (Naikare et al., 2006). Though a *feoB* mutant of *C. jejuni* NCTC 11168 had no growth disadvantage compared the wild-type strain during *in vitro* co-cultivation experiments, it showed a significant colonization defect in chickens and was outcompeted by the parental strain during a co-infection experiment with newborn piglets (Naikare et al., 2006). Future studies have to clarify how the puzzling diversity in the iron uptake systems affects the virulence and colonization fitness as well as the oxygen detoxification of different *C. jejuni* strains.

## A discreet tenant: the persistence of *C. jejuni* inside invaded epithelial cells

*C. jejuni* colonizes efficiently the gut of its diverse hosts where the majority of the population proliferates in the extracellular space of the mucus-filled intestinal crypts (Lee et al., 1986; Beery et al., 1988). Only a small fraction of the *C. jejuni* population can also be found inside intestinal epithelial cells when biopsies of infected humans (van Spreeuwel et al., 1985) or piglets (Babakhani et al., 1993) are analyzed. Similar to other facultative intracellular pathogens, like e.g., *S*. Typhimurium or *Legionella pneumophila*, *C. jejuni* resides during its intracellular stage inside a specific membrane-bound compartment, the so-called *Campylobacter*-containing-vacuole (CCV) (Watson and Galan, 2008). Though significant progress has been made in characterizing the metabolism of various facultative intracellular pathogens and in identifying bacterial factors facilitating efficient growth inside the host cells (Eisenreich et al., 2010), our knowledge about the metabolic properties that allow *C. jejuni* to persist in its intracellular compartment is still limited. In contrast to *S*. Typhimurium and *L. pneumophila*, *C. jejuni* does not multiply intracellularly (Watson and Galan, 2008). The absence of intracellular growth indicates that reduced metabolic activity might be sufficient for *C. jejuni* to maintain its membrane potential and viability inside its intracellular compartment. Accordingly, a metabolic downshift was recently described by quantitative proteome analysis of *C. jejuni* cells isolated from the CCVs of infected T84 colonic epithelial cells (Liu et al., 2012). This study showed that 20 h after infection 225 of the over 1400 detected *C. jejuni* proteins exhibit different levels compared to the proteome of *C. jejuni* cells isolated 2 h post-infection. Interestingly, 211 of the 225 proteins were found at lower levels, whereas only 14 proteins showed a 2- to 11-fold higher level, most prominently the acetyl-CoA synthetase ACS. The levels of the catalase KatA and the thiol peroxidase Tpx showed slightly but significant higher levels at 20 h compared to 2 h after infection. Surprisingly, KatA activity was not required by *C. jejuni* to survive within Hep-2 cells (Day et al., 2000), whereas the superoxide dismutase *sodB* mutant of *C. jejuni* showed abolished intracellular survival (Pesci et al., 1994; Novik et al., 2010). Interestingly, other proteins involved in oxidative stress response like AhpC, SodB, Trx and TrxB showed unaltered or slightly decreased levels, similar to proteins involved in the protection of *C. jejuni* against environmental stress (GroEL, DnaK, HtrA, GroES, GrpE, ClpB, DnaJ) (Liu et al., 2012).

The quiescent status of this pathogen inside the CCV is further enforced by the observation that many *C. jejuni* proteins with lower levels during the extended intracellular persistence are required for protein synthesis (Rps and Rpl proteins), the biosynthesis of amino acids (HisA, DapA, LysC, PheA), purines (PurH, PurL, PurM) and fatty acids (FabH, FabZ). In addition, several protein- and peptide-degrading proteins (ClpB, HslU, M16 and M24/M37 peptidase) and transporters involved in the uptake of amino acids (LivFJK, SdaC), phosphate (PstS) and metals like iron (Cj0175c), tungsten (TupA) or nickel (NikZ) showed lower levels 20 h post-infection (Liu et al., 2012). The metabolic downshift observed for intracellular *C. jejuni* is further reflected by decreased levels of proteins involved in the intermediary metabolism and gluconeogenesis like Tal, TpiA, GapA, FbaA and Pyk (Liu et al., 2012).

Most strikingly, *C. jejuni* undergoes a remarkable respiratory reprogramming when residing in its intracellular compartment for an extended time period: Proteins involved in the aerobic respiration like the cbb3-type cytochrome c oxidase subunits CcoO and CcoP and the ubiquinol-cytochrome c reductase (PetA, PetC) exhibited decreased protein levels after 20 h inside cultured epithelial cells. Such a decreased level was also seen for the formate dehydrogenase (FdhAB), the nitrate (NapAB) and nitrite (NrfA) reductases. *C. jejuni* seems to adapt its respiration mode to the oxygen-restricted conditions inside the CCV (Watson and Galan, 2008; Liu et al., 2012; Pryjma et al., 2012) by fumarate respiration: The fumarate reductase *frdA* mutant of *C. jejuni* 81–176 showed a significant intracellular survival defect inside COS-1 cells (Liu et al., 2012) and the importance of fumarate respiration was further supported by the reduced intracellular survival rate of an aspartate ammonia-lyase *aspA* mutant (Novik et al., 2010). In contrast to the *frdA* mutant, an *mrfA* mutant showed no decreased viability in the CCV after 20 h (Liu et al., 2012). *C. jejuni* 81–176 mutants with abolished formate (*fdhB*; *cjj81176_1502*), nitrate (*napG*; *cjj81176_0802*) and TMAO or other N- and S-oxide (*torA*; *cjj81176_0291*) respiration showed also no intracellular survival defect (Liu et al., 2012). Moreover, the cyanide-insensitive oxidase subunit CioA (CJJ81176_0118) with low affinity to oxygen is not required for the intracellular survival of *C. jejuni* 81–176 (Liu et al., 2012).

Additional insights into the intracellular lifestyle of *C. jejuni* provided microarray experiments analyzing the expression of *C. jejuni* 81–176 genes during the adhesion to and invasion into the human INT 407 cell line. This approach identified the stringent response regulator protein SpoT (Cj1272c) as being upregulated during the first 6 h post-infection of the cultured epithelial cells (Gaynor et al., 2005). SpoT modulates the guanosine tetraphosphate (ppGpp) metabolism in *C. jejuni* and inactivation of *spoT* in *C. jejuni* 81–176 resulted in a decreased synthesis of ppGpp similar as described for other bacterial pathogens (Dalebroux et al., 2010). The *spoT* mutant of *C. jejuni* 81–176 showed only a slight defect in adherence to and invasion into INT 407 cells, but a more pronounced defect in its intracellular survival over 20 h in comparison to the wild-type strain (Gaynor et al., 2005). Moreover, inactivation of *spoT* resulted in a decreased level of poly-phosphate (poly-P) in *C. jejuni* 81–176 comparable to a mutant strain lacking the polyphosphate kinase 1 (*ppk1*) mutant. PPK1 (Cj1359; CJJ81176_1361) uses ATP for the generation of poly-P, which participates in the stress response of *C. jejuni* and enables the survival during osmotic shock and low-nutrient stress. It was demonstrated that PPK1 is required for extended survival in infected INT407 cells (Candon et al., 2007), but as the *ppk1* mutation results, similar to the mutation of *spoT*, in pleiotropic effects, it does not allow the identification of mechanisms solely required for the intracellular survival. Poly-P might not only be important as a metabolic regulator and protector against various stresses like hydrogen peroxide and osmotic stress, it might also serve as an energy and phosphate source (Kornberg, 1995) that enhances the viability of *C. jejuni* while residing in the nutrient restricted environment of the CCV.

Though progress has been made in recent years to describe the nutrient requirements that allow *C. jejuni* to thrive in the gastrointestinal lumen of its hosts, how *C. jejuni* maintains its viability inside invaded epithelial cells and energizes its intracellular persistence remains ill-defined.

## Outlook

Campylobacteriosis of humans in industrialized countries is assumed to be an accidental and transitory event in the life style of *C. jejuni*. Such short-term interactions prevent co-evolution and potential fine-tuning of *Campylobacter* virulence factors. Consequently, the evolutionary forces that shape the metabolic and pathogenic properties of *C. jejuni* occur during the interactions with the microbiota of its primary hosts rather than within the human gastrointestinal ecosystem. Still, the metabolic framework of *C. jejuni* is sufficient to overcome the colonization resistance in the human gastrointestinal tract and to efficiently promote its proliferation, leading consequently to the development of diarrhea. These findings illustrate the necessity for future comprehensive studies to investigate the extent to which metabolic factors mediate the long-term persistence of *C. jejuni* in its natural hosts like chicken and cattle. Specifically targeting such growth-promoting metabolic pathways for the development of drugs might not only lower the colonization burden in animal hosts with *C. jejuni* but will consequently lower the exposure doses for humans. Moreover, the restricted carbohydrate catabolism of *C. jejuni* in comparison with other enteropathogenic pathogens like *S*. Typhimurium (Becker et al., 2006; Steeb et al., 2013) should facilitate the discovery of “metabolic Achilles heels” for the successful design of drugs against *Campylobacter*.

### Conflict of interest statement

The author declares that the research was conducted in the absence of any commercial or financial relationships that could be construed as a potential conflict of interest.
